# Identification of Anti-Tuberculosis Drugs Targeting DNA Gyrase A and Serine/Threonine Protein Kinase PknB: A Machine Learning-Assisted Drug-Repurposing Approach

**DOI:** 10.3390/tropicalmed9120288

**Published:** 2024-11-25

**Authors:** Dongwoo Lee, Md Ataul Islam, Sathishkumar Natarajan, Dawood Babu Dudekula, Hoyong Chung, Junhyung Park, Bermseok Oh

**Affiliations:** 1 Department of Biomedical Science, Graduate School, Kyung Hee University, Seoul 02447, Republic of Korea; dwlee@3bigs.com; 2 3BIGS Co., Ltd., B-831, Geumgang Penterium IX Tower, Hwaseong 18469, Republic of Korea; sathish@3bigs.com (S.N.); hychung@3bigs.com (H.C.); 3 3BIGS Omicscore Pvt., Ltd., 909 Lavelle Building, Richmond Circle, Bangalore 560025, India; ataul@3bigs.com (M.A.I.); dawood@3bigs.com (D.B.D.)

**Keywords:** tuberculosis, DNA Gyrase A, PknB, drug-repurposing, molecular docking, machine learning

## Abstract

Tuberculosis (TB) is a global health challenge associated with considerable levels of illness and mortality worldwide. The development of innovative therapeutic strategies is crucial to combat the rise of drug-resistant TB strains. DNA Gyrase A (GyrA) and serine/threonine protein kinase (PknB) are promising targets for new TB medications. This study employed techniques such as similarity searches, molecular docking analyses, machine learning (ML)-driven absolute binding-free energy calculations, and molecular dynamics (MD) simulations to find potential drug candidates. By combining ligand- and structure-based methods with ML principles and MD simulations, a novel strategy was proposed for identifying small molecules. Drugs with structural similarities to existing TB therapies were assessed for their binding affinity to GyrA and PknB through various docking approaches and ML-based predictions. A detailed analysis identified six promising compounds for each target, such as DB00199, DB01220, DB06827, DB11753, DB14631, and DB14703 for GyrA; and DB00547, DB00615, DB06827, DB14644, DB11753, and DB14703 for PknB. Notably, DB11753 and DB14703 show significant potential for both targets. Furthermore, MD simulations’ statistical metrics confirm the drug–target complexes’ stability, with MM-GBSA analyses underscoring their strong binding affinity, indicating their promise for TB treatment even though they were not initially designed for this disease.

## 1. Introduction

Tuberculosis (TB) is a transmissible and serious illness with significant global morbidity and mortality [1]. Despite concerted control and eradication efforts, TB continues to be one of the most ancient and lethal infectious diseases worldwide [2]. Before COVID-19, a single infectious agent, TB, was on the top of killing humans and also above the rank of human deficiency virus (HIV) [3]. According to the World Health Organization (WHO), TB killed about 1.3 million in 2022, of which 167,000 were HIV co-infected individuals. In the year 2022, more than 10 million people were infected by TB globally in men, women, and children 5.8, 3.5, and 1.3 million, respectively. The above explosive data indicate that the situation remains severe as of 2024 without significant intervention and improvement. Low- and middle-income countries are more prone to TB, with a significant concentration in Southeast Asia and Africa [4]. Specifically, India, Indonesia, China, the Philippines, Pakistan, Nigeria, and South Africa are among the countries with the highest TB burden, with approximately account for about 60% of global TB deaths [5].

The causative agent of TB is *Mycobacterium tuberculosis* (*Mtb*) [6]. This slow-growing, acid-fast bacillus primarily targets the lungs, causing pulmonary TB, but can also lead to extrapulmonary TB. The efficacy of traditional antibiotics is diminishing due to the emergence of resistant pathogens. Such resistance complicates treatment, prolongs illness, escalates medical expenses, and heightens mortality rates. Therefore, there is a pressing need for extensive research and development aimed at discovering novel therapies to counter resistance, enhance efficacy, and adopt a more holistic approach to managing infectious diseases.

DNA Gyrase (Gyr) facilitates the supercoiling of DNA, which is crucial for various DNA transactions, including replication, transcription, and repair [7]. The inhibition of DNA gyrase disrupts these processes, leading to bacterial cell death [8]. Gyr, comprising the subunits GyrA and GyrB, is a crucial enzyme for the viability and proliferation of *Mtb*, making it an attractive target for TB drug discovery [9]. GyrA is primarily responsible for DNA cutting and re-ligation during the supercoiling process, whereas GyrB provides the necessary ATPase activity that powers the supercoiling process executed by GyrA. Inhibiting GyrA disrupts the bacterial DNA’s structural integrity, leading to blocked replication and transcription and, consequently, bacterial cell death [10]. Consequently, GyrA is a crucial enzyme for therapeutic intervention in TB treatment.

Serine/threonine protein kinase (STPKs) represent a set of 11 vital targets crucial for transmembrane signaling [11]. Out of all, three targets, such as PknA, PknB, and PknG, have already been validated and approved for anti-TB drug development [12]. PknA and PknB are essential for mycobacterial growth, whereas PknG is important in promoting mycobacterial survival inside host cells and essential to *Mtb* virulence. PknG plays a crucial role in enhancing mycobacterial survival within host cells. PknA and PknB are critically involved in regulating key cellular processes, such as cell growth, division, and metabolism, making them indispensable for the virulence of *Mtb* [13]. Already, PknB has been explored on a large scale as an *Mtb* target to develop potential anti-TB molecules that can be substantiated by reported PknB inhibitors [14] such as mitoxantrone [15], staurosporine, and its derivatives [16,17], several aminopyrimidine derivatives [18,19,20,21], compound IMB-YH-8 [22,23,24], (E)-4-oxo-crotonamide derivatives [25,26], 8H-pyrido [2,3-d]pyrimidin-7-one24, [27,28], thiophene amide derivatives [28], 2-aminopurine [29], N-phenylmethylindole-2-carboxamide [30], and 4-(1H-imidazo [4,5-c]pyridin-2-yl)-1,2,5-oxadiazol-3-amine [31]. Hence, the strategic focus on PknB could lead to the development of new, more effective anti-TB therapies, addressing the urgent need for innovative treatment options in the face of rising drug resistance. Therefore, further research into PknB inhibitors offers significant potential for advancing TB drug discovery.

Given their essential role in bacterial survival, GyrA and PknB continue to be promising focus areas for TB drug development, addressing the urgent need for novel and effective treatment options. Despite significant advancements in science and technology, the drug development process remains slow, time-consuming, and trial-error-based approach [32]. It has been reported that converting a small molecule into a drug may take approximately 15 years and about 2 billion USD in investment [33]. Clinical studies of new drugs are the most expensive stage in the drug discovery pipeline. In addition to the above, the high failure rate in clinical trials, which is currently 90%, can largely be attributed to issues originating in the early stages of discovery, such as inadequate target validation and suboptimal ligand properties [34]. Identifying rapid and accessible methods to discover more diverse pools of high-quality chemical probes, hits, and leads with optimal absorption, distribution, metabolism, excretion, and toxicology (ADMET) and pharmacokinetics (PK) profiles during the early stages of drug discovery and development (DDD) would enhance outcomes in preclinical and clinical studies, ultimately facilitating the development of more effective, accessible, and safer drugs. Among several pivotal computer-assisted drug discovery approaches, drug repurposing (also known as drug repositioning) can be an excellent pioneer method of finding drug potential candidates while circumventing several experimental methodologies [35]. Drug repurposing is a method in which the existing approved drugs can be used for a disease for which it was not invented [36]. Drug repurposing became an excellent choice for academia and industries due to its advantages, including low-risk factors as already found safe in pre-clinical and human trials, less time for development due to most of the experimental assessments, including formulation already completed, and drastic reduction of the development investment [37].

The present investigation sought to exploit drug repurposing to discover drug candidates for TB targeting GyrA and PknB. This objective was achieved through a combination of established computational methodologies, including similarity search, multi-tier molecular docking, machine learning (ML)-based binding free energy calculations, and molecular dynamics (MD) simulations. The study’s validity was supported by the identification of several FDA-approved drugs with high potential against significant TB targets like GyrA and PknB.

## 2. Materials and Methods

### 2.1. Selection of GyrA and PknB Structure and Preparation

Of several available crystal structures, PDB IDs: 5BS8 [38] and 1O6Y [39] were selected as the final protein three-dimensional (3D) coordinates for GyrA and PknB, respectively. The resolution and difference between R-factor and R-free were found to be 2.40 Å and 0.035, and 2.20 Å and 0.039 for GyrA and PknB, respectively. Both the structures were developed through the X-ray diffraction method. Structural data of both structures have already been published in peer-reviewed journals. The total number of amino acid sequences identified is 503 in GyrA and 299 in PknB. The above two crystal structures were prepared using the Autodock tool (ADT) for further study. First of all, co-crystal water molecules, ions, unwanted protein chains, and heteroatoms were removed. Further, the missing atoms were detected and repaired, and hydrogens and Gasteiger charges were added, followed by the Autodock4 (AD4) atom type assigned. Finally, both the structures were saved in .pdbqt format.

### 2.2. Collection and Preparation of FDA-Approved Drugs

The entire set of FDA-approved drugs was collected from DrugBank [40] in a simplified molecular entry line system (SMILES) format with about 2500 drug candidates. The preparation of small molecules is critical for accurate molecular docking studies. Stabilizing the small molecule in a low-energy conformation is necessary to reflect its natural state. Proper protonation states at relevant pH conditions are vital for charge distribution and hydrogen bonding, preventing unrealistic binding scenarios. The OpenBabel [41] is an open-source, powerful tool for converting chemical file formats, adding hydrogens, optimizing geometries, and generating 3D coordinates and conformers. The FDA-approved drug molecules were prepared using the OpenBabel v3.1.1. In particular, each SMILES representation of the drug molecules generated the 3D conformers. The best conformer of each molecule was considered, and added hydrogens and Gasteiger charge [42]. Further, the protonation state of all molecules was maintained at physiological pH. For the molecular docking study, all the above molecules were saved in .pdbqt and .mol2 formats.

### 2.3. Fingerprint Based Similarity Search

Fingerprint-based molecular similarity search plays a pivotal role in the process of drug repurposing, which involves finding new therapeutic uses for existing drugs [43]. To perform the similarity search, a total of 30 TB drugs were considered query molecules. The DrugBank database was extensively explored to identify drugs used in antibacterial treatments. The study focused on drugs that have received approval for treating bacterial infections. The DrugBank ID and SMILES representation of the 30 TB drugs is given in Table S1 (Supplementary Materials). The entire set of FDA-approved drug molecules, except TB drugs, was used as the target database in the similarity search. Both sets of TB and FDA-approved drugs were used as SMILES to generate the fingerprints using the extended connectivity fingerprints4 (ECFP4). ECFP fingerprints are circular molecular fingerprints that encode the presence of substructures centered around each atom in a molecule [44]. These fingerprints are created iteratively, including neighboring atoms up to a specified radius. Specifically, ECFP4 uses a radius of 2 (covering up to four bonds from each atom) to generate the fingerprint [45]. Python RDKit [46] is a powerful open-source cheminformatics toolkit used to perform ECFP4 similarity searches. Each of the TB drugs was considered to check the similarity with each of the non-TB FDA-approved drugs with ECFP4 functionality, and the Tanimotto coefficient [47] was calculated. The Tanimoto coefficient is a statistical measure used to compare the similarity between two molecules and ranges from 0 to 1, indicating no similarity to an exact match, respectively [47]. Molecules found to have high similarity scores with TB drugs were further considered for the next level of analysis.

### 2.4. Molecular Docking Using Autodock Vina and PLANTS

Molecular docking plays a crucial role in drug repurposing by predicting the binding interactions between known drugs and target proteins associated with different diseases [48]. Drug molecules retained after the similarity search were considered for two tiers of molecular docking studies to explore the binding affinity towards GyrA and PknB. The molecular docking of the considered molecules was docked using Autodock vina (ADV) v.1.2.5 [49] and Protein-Ligand ANTSystem v.1.2 (PLANTS) [50]. ADV combines empirical scoring functions with a gradient-based optimization algorithm to efficiently explore the binding space and predict ligand–protein interactions. It uses an empirical scoring function to evaluate the binding affinity of each predicted binding pose. PLANTS employs an ant colony optimization algorithm to explore the ligand conformational space and predict the binding modes of ligands to protein targets. The active site coordinates for GyrA were determined to be (32.48, 27.54, 28.12) along the x-, y-, and z-axes, respectively, while the coordinates for PknB were (84.54, 87.55, 22.41). To incorporate the entire active site of GyrA, a grid size of 50 × 60 × 50 along the x-, y-, and z-axes was selected. For PknB, a grid size of 45 × 60 × 55 along the x-, y-, and z-axes was estimated suitable to cover the active site adequately. The above active site coordinates and grid size were used to dock the drug molecules in GyrA and PknB targets, and the binding energy was recorded from ADV and PLANTS.

### 2.5. Absolute Binding Affinity Using KDeep

The absolute binding affinity refers to the strength of the interaction between a ligand and a protein target in a biological system [51]. The absolute binding affinity of each drug molecule retained after the similarity search was considered for the absolute binding affinity calculation using the ML-based KDeep tool [52]. KDeep is one of the recent ML-assisted absolute binding affinity tools that is based on 3D-convolutional neural networks and it can be accessed through https://www.playmolecule.com/Kdeep/ (accessed on 18 April 2023). The tool accepts the best-docked pose in structural data format (.sdf) and target structure in .pdb format. It calculates the absolute binding affinity in kcal/mol unit on a successful run. Molecules retained after the similarity search were used to calculate the absolute binding affinity.

### 2.6. Molecular Dynamics Simulation

The drug molecules found with high binding affinity towards GyrA and PknB through ADV, PLANTS, and KDeep were considered to explore the stability at the active site cavity in dynamic states through all-atoms MD simulation using GROningen MAchine for Chemical Simulations (GROMACS) v2021.3 [53,54]. MD simulation is a powerful computational technique used to study dynamic behavior and molecular interactions. It also provides valuable insights into the stability, conformational changes, and binding dynamics of the complex over time. The topology of both GyrA and PknB was generated using the Chemistry at Harvard Macromolecular Mechanics (CHARMM36) [55] forcefield, whereas the small molecule topology was obtained using the SwissParam [56], an online topology generation tool (http://swissparam.ch/, accessed on 28 April 2023). The protein–ligand complexes were confined into the cubic box and solvated with the TIP3P [57] water model. A distance of 10 Å was maintained between the biological molecules and the wall of the box. The periodic condition was implemented during the simulation. The required quantity of Na^+^/Cl^−^ ions was added to neutralize each system. The energy minimization for a maximum of 50,000 steps was carried out to overcome the close contacts and overlapping in the molecular systems. Before the final production execution, the two levels of equilibration, including the isothermal−isochoric (NVT) ensemble and the isothermal−isobaric (NPT) ensemble, were performed to ensure uniform distribution of the solvent and ions. The system temperature was regulated at 300 K using a velocity-rescaling thermostat, while the reference pressure was maintained at 1 bar with the Berendsen barostat. The bond length constraints were imposed using the LINCS algorithm [58]. The short-range van der Waals cutoff was set to 1.2 nm, and the electrostatic long-range interactions were carried out with a particle-mesh Ewald scheme (PME) [59] with a grid spacing equal to 0.16 nm. Followed by the equilibration, each system from both GyrA and PknB was executed for 10 ns of MD simulation production. To explore the stability and dynamicity of small molecules inside the receptor protein, a number of statistical parameters from the MD simulation trajectories were calculated. These statistical parameters included protein backbone and ligand root-mean-square deviation (RMSD), amino acid root-mean-square fluctuation (RMSF), radius of gyration (RoG), and inter-molecular hydrogen bonds. Further, the free energy landscape (FEL) was calculated using MD DaVis [60] by considering the backbone RMSD and RoG. The FEL enlightens the conformational changes in terms of free energy.

### 2.7. Binding Free Energy Calculation Using MM-GBSA Approach

Binding free energy calculations using the molecular mechanics/generalized born surface area (MM-GBSA) method are prevalent in computational chemistry for assessing ligand–protein binding strength. The binding free energy (∆Gbind) derived from MM-GBSA is typically viewed as more dependable than molecular docking. 

The gmx_MMPBSA v.1.6.2 [61] package was used to calculate the ∆Gbind for each of the final drug molecules against GyrA and PknB. A total of 2000 frames out of 10,000 with an interval of 5 were considered for each simulation trajectory.

Following expressions are used to calculate the ΔGbind.

(1) $$\Delta G_{\mathrm{bind}}=\langle G_{\mathrm{complex}}\rangle -\langle G_{\mathrm{receptor}}\rangle -\langle G_{\mathrm{ligand}}\rangle$$

Here, G_complex_, G_receptor_, and G_ligand_ represent the binding energies of the protein–ligand complex, receptor, and ligand, respectively.

The ΔG_bind_ can also be expressed as

(2) $$\Delta G_{\mathrm{bind}}=\Delta H\;-\;T\Delta S$$

The enthalpy of binding, represented as ∆H, defines the binding heat, while T∆S signifies the change in conformational entropy post-ligand binding. Upon excluding the entropic component, the resultant value denotes the effective free energy, which adequately allows for the comparison of small molecule binding energies. Further, the ∆H can be split into the following individual terms

(3) $$\Delta H={\Delta E}_{\mathrm{MM}}{+\Delta G}_{\mathrm{sol}}$$

where ΔE_MM_ can be expressed as a summation of bonded and non-bonded terms as below.

(4) $${\Delta E}_{\mathrm{MM}}{=\Delta E}_{\mathrm{bonded}}{+\Delta E}_{\mathrm{nonbonded}}$$

The term E_bonded_ encompasses bond stretching, angle bending, and torsion angle contributions, while ∆E_nonbonded_ combines the electrostatic and van der Waals interactions. The specific expressions for these terms are provided below.

(5) $${\Delta E}_{\mathrm{bonded}}{=\Delta E}_{\mathrm{bond}\_\mathrm{length}}{+\Delta E}_{\mathrm{angle}}{+\Delta E}_{\mathrm{dihedral}}$$

(6) $${\Delta E}_{\mathrm{nonbonded}}{=\Delta E}_{\mathrm{ele}}{+\Delta E}_{\mathrm{vdW}}$$

In generalized Born (GB) models, the solvation energy (∆G_sol_) can be computed based solely on the polar component. The nonpolar (NP) component is commonly assumed to be linearly related to the molecule’s total solvent-accessible surface area (SASA), with a scaling factor determined from experimental solvation energies of small, nonpolar compounds. The solvation energy and nonpolar energy terms are expressed below.

(7) $${\Delta G}_{\mathrm{sol}}{=\Delta G}_{\mathrm{polar}}{+\Delta G}_{\mathrm{non}-\mathrm{polar}}{=\Delta G}_{\mathrm{GB}}{+\Delta G}_{\mathrm{non}-\mathrm{polar}}$$

(8) $${\Delta G}_{\mathrm{non}-\mathrm{polar}}{=\mathrm{NP}}_{\mathrm{TENSION}}{+\Delta }_{\mathrm{SASA}}{+\mathrm{NP}}_{\mathrm{OFFSET}}$$

After successfully computing the binding free energy (∆G_bind_) for each molecule, the results were recorded together with the corresponding standard deviation.

## 3. Results and Discussion

**Figure 1:**
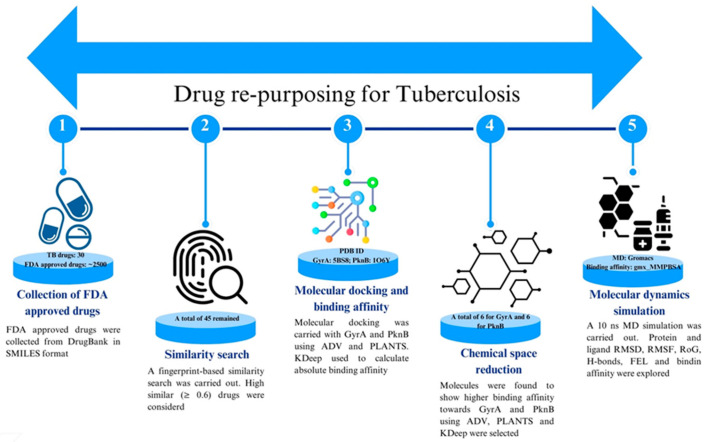
Workflow of the drug-repurposing for GyrA and PknB.

A comprehensive drug-repurposing strategy was implemented to identify potential drug candidates for TB. This approach integrated various computational techniques, including fingerprinting-based similarity search, multi-tier molecular docking, ML-guided absolute binding affinity prediction, and all-atoms MD simulation. The workflow of the study, depicted in Figure 1, showcases the step-by-step process followed to analyze and valuate the potential drug molecules for TB treatment.

### 3.1. Similarity Search

Using a Python RDKit ECFP4-assisted similarity search, 30 TB drugs were systematically compared with all non-TB, FDA-approved drugs in the database to assess potential matches and similarities based on the Tanimoto coefficient. To identify drug molecules similar to known TB medications, compounds with a Tanimoto coefficient ≥ 0.6 were chosen for further investigation. Among FDA-approved drugs, 45 compounds closely resembled TB drugs. Details, including SMILES representation and DrugBank ID for each drug, are in Table S2. These findings highlight potential candidates from existing FDA-approved drugs that may offer new avenues for repurposing.

### 3.2. Screening Through Molecular Docking and Absolute Binding Affinity

**Figure 2:**
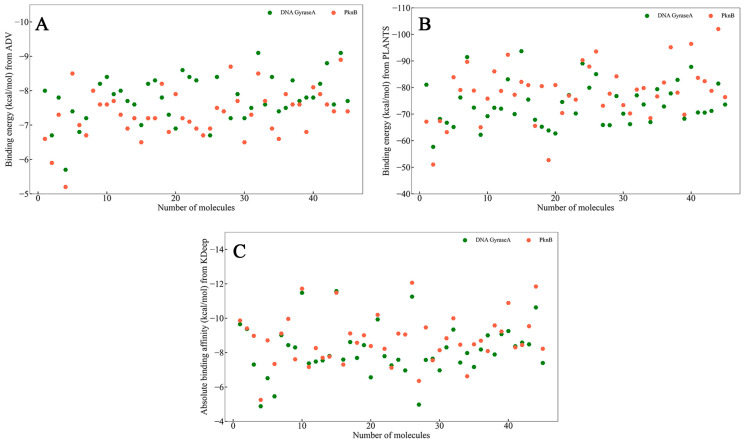
The binding energy of selected 45 molecules from similarity search. (**A**) Binding energy using ADV, (**B**) binding energy using PLANTS, and (**C**) binding affinity from KDeep. Green: DNA Gyrase, Orange: PknB.

Drug candidates from the initial similarity search underwent molecular docking studies with ADV and PLANTS, specifically targeting TB-related proteins GyrA and PknB. During this process, the most favorable docking pose for each molecule was selected for further analysis. These selected poses were then utilized to calculate the absolute binding affinity using the KDeep tool. The resulting binding energies obtained from ADV (Figure 2A), PLANTS (Figure 2B), and KDeep (Figure 2C) were meticulously analyzed to refine and narrow down the chemical space, focusing on the most promising drug candidates. At the initial stage of the study, the top 20 molecules for each of the targets, GyrA and PknB, were selected based on their binding energies derived from ADV, PLANTS, and KDeep tools. This selection was conducted separately for each docking tool and molecular target. Consequently, a total of 60 distinct molecules were identified for both GyrA and PknB. This comprehensive selection process ensured that the most promising candidates were considered for further investigation, as determined by their binding affinities calculated through multiple independent methods.

The drug candidates that exhibited high binding affinities across all three computational tools, such as ADV, PLANTS, and KDeep, were evaluated. Within this cohort of screened molecules, six drug candidates for each target, GyrA, and PknB, consistently ranked within the top 20 based on their binding energy metrics obtained from all three assessment platforms. This convergence of high-affinity scores across multiple docking and binding affinity prediction tools highlights these selected drug candidates’ robustness and potential efficacy in targeting GyrA and PknB for TB treatment. The six drug molecules demonstrating high binding affinities for GyrA were DB00199, DB01220, DB06827, DB11753, DB14631, and DB14703. Similarly, for PknB, the six drug molecules were DB00547, DB00615, DB06827, DB14644, DB11753, and DB14703. These selected drug candidates exhibited a diverse array of pharmacophoric features and significant functional groups, which likely contribute to their potential to form robust interactions within the active sites of their respective targets. The varied pharmacophoric characteristics and functional groups present in these molecules may enhance their binding specificity and stability, thereby increasing the efficacy of these drug candidates in inhibiting GyrA and PknB. This diversity in structural features also helps overcome resistance mechanisms and improve overall therapeutic outcomes. It is noteworthy that three drug molecules, DB06827, DB11753, and DB14703, were identified as common candidates for both GyrA and PknB targets. This dual-target efficacy suggests that these molecules possess key structural and functional properties, enabling them to interact effectively with the active sites of both proteins. The overlapping occurrence of these drug candidates signifies their potential as broad-spectrum inhibitors, which could simultaneously target multiple pathways involved in TB pathogenesis. This dual interaction capability may make them particularly valuable for therapeutic strategies to minimize drug resistance and treatment efficacy. The binding energy of the top selected drug molecules for GyrA and PknB from ADV, PLANTS, and KDeep is given in Table 1. High negative binding energy from all three tools undoubtedly exerted the potentiality of the selected drug candidates towards GyrA and PknB.

**Figure 3:**
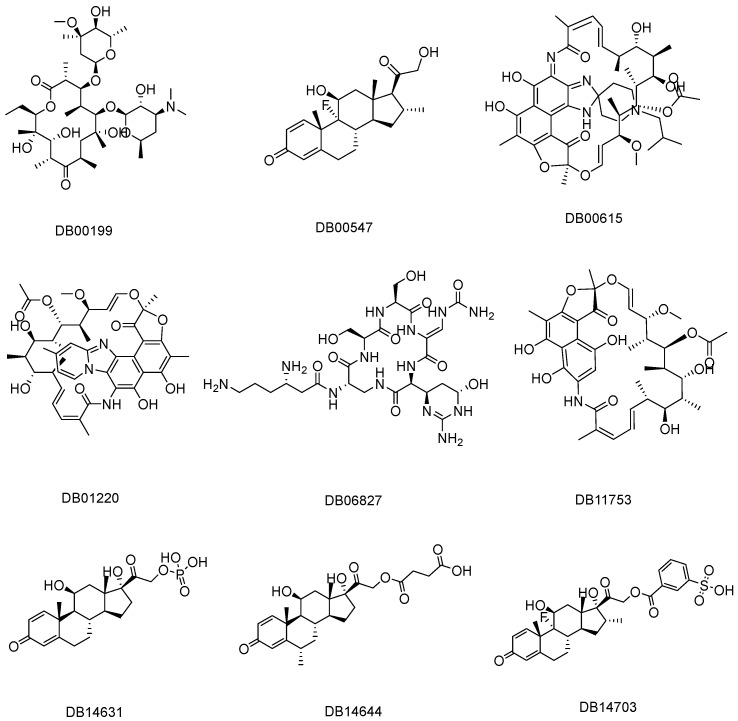
A two-dimensional representation of the final selected drugs for GyrA (DB00199, DB01220, DB06827, DB11753, DB14631, and DB14703) and PknB (DB00547, DB00615, DB06827, DB14644, DB11753, and DB14703).

A comprehensive two-dimensional representation of the final selected drug candidates is provided in Figure 3. This figure illustrates the molecular structures of the selected drugs, detailing their various functional groups and pharmacophoric features. By visually assessing these structures, one can discern the specific chemical attributes that may contribute to their binding efficacy and interaction specificity with the target sites, GyrA and PknB. The illustration in Figure 3 thus serves as a critical reference for understanding the molecular architecture and comparative structural analysis of these promising drug candidates.

### 3.3. Binding Interactions Profile

**Figure 4:**
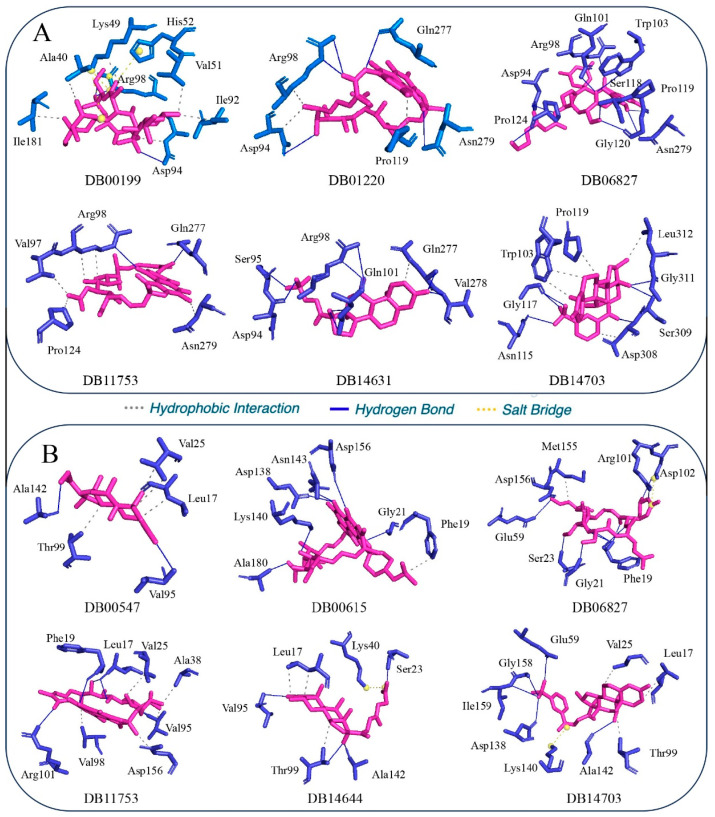
Binding interactions profile of the selected drug molecule with (**A**) GyrA and (**B**) PknB. Pink: Drug candidates, Blue: Targets.

The three-dimensional representations of the binding interactions are depicted in Figure 4A,B for GyrA and PknB, respectively. Additionally, the specific amino acids involved in these interactions with each target protein are detailed in Table 2. Analysis of the interaction profiles revealed that nearly all drug molecules engaged with their respective targets through a combination of hydrogen bonds and hydrophobic interactions. Moreover, specific drug–target pairs exhibited the formation of salt bridges, namely, DB00199 with GyrA and DB06827, DB14644, and DB14703 with PknB. These salt bridges suggest an additional layer of interaction stability and specificity, which could be critical for effectively inhibiting the target proteins.

#### 3.3.1. Binding Interaction with DNA GyrA

Detailed analysis of the binding interactions of DB00199 with GyrA revealed the significance of various functional groups in the molecule’s interaction profile. Specifically, the methyl group at the methyloxane moiety of DB00199 was crucial for establishing hydrophobic interactions with the residues Val51 and Ile92. Additionally, the hydroxyl and oxo groups on the larger ring structure were found to form critically hydrogen bonds with Asp94 and Arg98. Beyond these interactions, hydrophobic contacts with GyrA were further stabilized by interactions with Ala40, Lys49, Asp94, and Ile181, indicating the drug molecule’s comprehensive engagement with multiple hydrophobic protein regions. Furthermore, the interaction profile was enhanced by the formation of three salt bridges between DB00199 and the residues Lys49, His52, and Arg98 of GyrA. These salt bridges likely contribute to the overall binding stability and specificity of DB00199 to GyrA, thus highlighting its potential as a potent inhibitor. A comprehensive interaction analysis of DB01220 with GyrA uncovered numerous hydrogen bonds and hydrophobic interactions contributing to the drug’s binding affinity and specificity. Among the key interactions, the amino acid residues Asp94 and Arg98 of GyrA were revealed to be crucial, as they formed both hydrogen bonds and hydrophobic interactions with DB01220, significantly stabilizing the drug–protein complex. The residues Gln277 and Asn279 were also identified as forming important hydrogen bonds with DB01220, further securing its binding orientation and enhancing interaction stability. Pro119 contributed to the binding through hydrophobic interactions, complementing the hydrogen bonding network and providing additional stabilization. The combined effect of these interactions highlights the robust binding potential of DB01220 to GyrA, positioning it as a promising candidate for further exploration in tuberculosis treatment. The binding interactions of the drug molecule DB06827 with GyrA were found to be exclusively through hydrogen bonds involving several key amino acid residues. Specifically, DB06827 formed hydrogen bonds with Asp94, Arg98, Gln101, Trp103, Ser118, Pro119, Gly120, Pro124, and Asn279. These interactions suggest a strong and highly specific binding affinity, mediated primarily through polar interactions, that could significantly contribute to the stability of the drug–protein complex. In contrast, the interaction profile of another important drug molecule, DB11753, with GyrA exhibited a combination of hydrogen bonds and hydrophobic interactions. Hydrogen bonds were formed with the residues Arg98 and Gln277, reinforcing the binding orientation and specificity. Additionally, DB11753 engaged in hydrophobic interactions with Val97, Arg98, Pro124, and Asn279. This dual mode of interaction through both hydrogen bonding and hydrophobic contacts suggests that DB11753 is well positioned to establish a robust and stable association with GyrA, enhancing its potential efficacy as an inhibitory agent. A detailed examination of the interactions between DB143631 and GyrA identified a set of eight critical amino acid residues that play a significant role in the binding process. Specifically, the residues Asp94, Ser95, Arg98, Gln101, Gln277, and Val278 were found to form potential hydrogen bonds with DB143631. These hydrogen bonds are integral to the stabilization of the drug–protein complex, ensuring a strong and specific interaction. Furthermore, among these residues, Gln277 also engaged in hydrophobic interactions with DB143631. This indicates a dual role for Gln277 in reinforcing the binding affinity through both polar and nonpolar contacts. The combination of multiple hydrogen bonds and a hydrophobic interaction showed the comprehensive binding strategy employed by DB143631, making it a promising candidate for effective inhibition of GyrA in tuberculosis treatment. A thorough analysis of the binding interactions between DB14703 and GyrA revealed crucial insights into the molecular interaction profile. Notably, the residue Asp308 of GyrA emerged as a pivotal component, engaging in both hydrogen bond formation and hydrophobic interactions with DB14703. This dual interaction role expresses the significance of Asp308 in stabilizing the drug within the active site, highlighting its importance in the binding mechanism. Beyond the critical role of Asp308, several other residues were identified as key players in the interaction landscape of DB14703 with GyrA. The residues Ala115, Gly117, Ser307, and Gly311 were found to form essential hydrogen bonds with DB14703. Additional residues, including Trp103, Arg119, and Leu312, were observed to participate in hydrophobic interactions with DB14703. The involvement of hydrophobic interactions suggests that these residues play a key role in maintaining the spatial conformation of DB14703 within the GyrA binding site. The combination of hydrogen bonds and hydrophobic interactions not only secures drug molecules within the active site of GyrA but also ensures the necessary conformational stability required for effective inhibition. This intricate network of interactions delineates the drug candidates as a robust candidate for GyrA inhibition, providing a promising foundation for its development in anti-TB therapies. These findings signify the importance of a multifaceted binding strategy in achieving effective and durable drug–target interactions. Upon close inspection of the interaction profiles, it becomes evident that several amino acid residues are recurrently involved in binding with multiple drug molecules. This observation indicates a comparable pattern of binding interactions among the proposed drug candidates. Notable residues such as Asp94, Arg98, Gln101, and Gln277 frequently participate in hydrogen bonding and hydrophobic interactions across different drug molecules, highlighting their critical roles in binding. The consistent involvement of these amino acids suggests that the drug molecules may share a common interaction mechanism with the target protein, GyrA.

#### 3.3.2. Binding Interactions with PknB

Figure 4B illustrates the binding interaction profiles between the proposed drug molecules and PknB. A comprehensive analysis of these interactions reveals that all the molecules exhibited a significant and diverse array of potential binding interactions with PknB. The visualization provided in Figure 4B showcases the intricate network of hydrogen bonds, hydrophobic interactions, and potentially other key interactions that contribute to the binding affinity and specificity of the drug candidates towards PknB. These interactions play a crucial role in determining the efficacy and potency of the molecules as inhibitors of PknB. Overall, the detailed binding interaction profile presented in Figure 4B highlights the promising therapeutic potential of the proposed drug molecules against PknB in the context of combating TB. This comprehensive understanding of the molecular interactions paves the way for further optimization and development of these compounds as viable treatment options for tuberculosis. In the investigation of molecular interactions between PknB and specific drug molecules, Val95 of PknB emerged as a key residue engaged in distinct binding interactions. Val95 formed hydrogen bonds with DB00547 and DB14644, as well as hydrophobic interactions with DB11753, underlining its versatile role in mediating diverse molecular contacts. Amino acid Val25 also played a significant role by establishing crucial hydrophobic interactions with DB00547 and DB14703, highlighting the importance of nonpolar interactions in the binding process. Furthermore, the involvement of Ala142 was pivotal in facilitating hydrogen bonds between PknB and DB00615, DB14644, and DB14703, emphasizing its mediation of polar interactions critical for stabilizing the drug–protein complexes. The pivotal role of Asp138 was unveiled as essential for interactions with DB00615, DB11753, and DB14703, revealing its significance in facilitating multiple molecular contacts crucial for binding affinity. Furthermore, the binding profile highlighted the critical involvement of Lys140 in mediating interactions with the drug molecules. Lys140 not only formed a hydrogen bond with DB00615 but also played a key role in establishing salt bridges with DB14644 and DB14703, illustrating the diverse nature of its interactions and its contribution to the stability of the complexes. Additionally, including nonpolar residues Leu17 and the methyl group of Thr99 emerged as key determinants in forming essential hydrophobic interactions with DB00547, DB14644, and DB14703. Phe19 emerged as a crucial player, engaging in both hydrophobic and hydrogen bond interactions with DB00615, DB06827, and DB11753. These dual interactions portrayed the versatile nature of Phe19 in mediating distinct molecular contacts essential for binding specificity. Similarly, Gly21 and Asp156 played critical roles by forming hydrogen bond interactions with DB00615 and DB06825. The participation of these residues highlighted their involvement in establishing key polar interactions crucial for stabilizing the drug–protein complexes. Furthermore, Arg101 of PknB demonstrated a robust affinity by strongly forming hydrogen bonds with DB06827 and DB11753, emphasizing its essential role in mediating specific molecular interactions essential for binding stability. Ser23 was identified as crucial, forming hydrogen bonds with both DB06827 and DB14644, indicating its consistent involvement in establishing polar interactions with distinct drug molecules. Similarly, Glu59 exhibited shared interactions with DB06827 and DB14703, emphasizing its role in mediating specific molecular contacts critical for binding specificity. Additionally, Asp102 demonstrated a multipurpose binding profile by forming dual bonds, such as a hydrogen bond and a salt bridge with DB06827, showcasing its ability to engage in diverse interactions crucial for the stability of the drug–protein complex. Furthermore, other key residues, including Asn143 and Ala180, Thr99, Gly158, and Ile159, were instrumental in interacting with DB00615, DB14644, and DB14703, respectively. Met155, Val25, and Val98 were identified as essential players in interacting with DB06827 and DB11753 through potential hydrophobic interactions. Met155’s role in mediating hydrophobic contacts with DB06827 emphasized its contribution to the nonpolar interactions crucial for stabilizing the drug–protein complex. Similarly, Val25 and Val98 demonstrated critical involvement in establishing hydrophobic interactions with DB06827 and DB11753, respectively, highlighting their significance in development the hydrophobic contacts necessary for binding affinity. The clear in-depth analysis of the binding interactions profile between the proposed drug molecules and PknB demonstrates a robust and significant number of binding interactions. The comprehensive examination reveals diverse interactions, including hydrogen bonds, hydrophobic interactions, salt bridges, and other critical contacts between the drug and receptor molecules. These findings collectively indicate a robust binding affinity between the proposed drug compounds and PknB. These interactions’ consistent and varied nature highlights the targeted and effective molecular mechanisms through which the drug molecules interact with PknB, emphasizing their potential as potent inhibitors. The conclusive evidence from these binding interaction analyses reinforces the notion of a high affinity and specific molecular recognition between the proposed drug molecules and the PknB receptor, laying a solid foundation for their potential efficacy in targeted therapy against TB.

**Figure 5:**
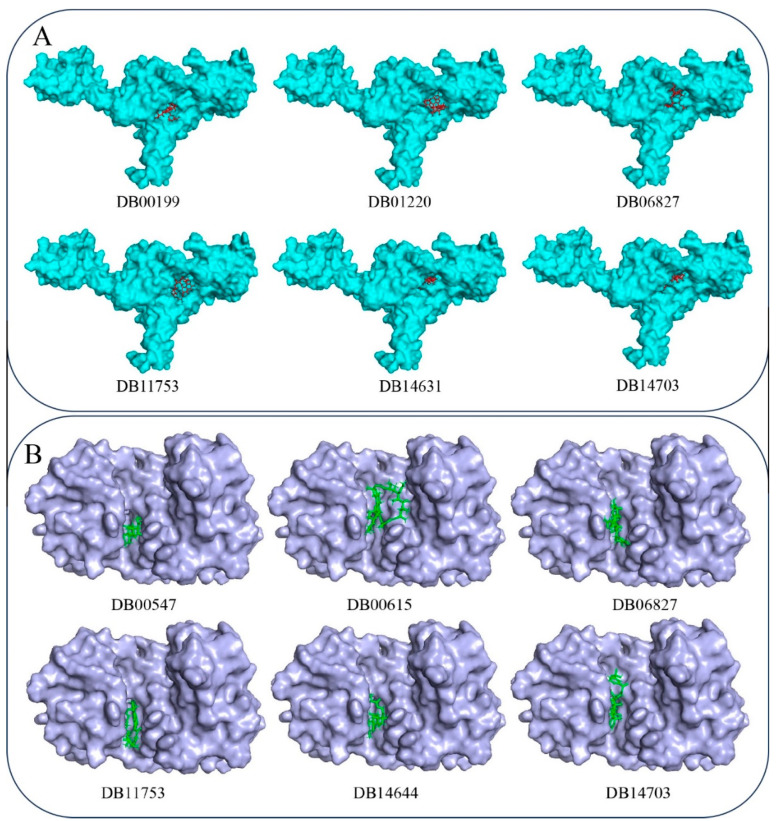
Binding mode proposed drug molecules at (**A**) GyrA and (**B**) PknB.

The binding mode of the respective drug molecules was explored, and the findings are visually represented in Figure 5. The illustrative depiction in Figure 5 explicitly portrays that all the drug molecules seamlessly fit within the receptor cavity, showcasing a comfortable and precise binding mode. The visual representation ensures the optimal positioning of the molecules within the binding site of the receptor, suggesting a strong and favorable interaction geometry. The clear and detailed visualization in Figure 5 is compelling evidence that the proposed drug compounds engage with the receptor to ensure a snug and effective binding configuration. This alignment further supports the notion of a high-affinity interaction and a promising therapeutic potential for the drug molecules in targeting PknB for TB treatment.

### 3.4. Molecular Dynamics Simulation

From the MD simulation trajectories, the maximum, minimum, and average values of the above parameters were calculated and given in Table 3.

#### 3.4.1. Root-Mean-Square Deviation

##### Protein Backbone RMSD

**Figure 6:**
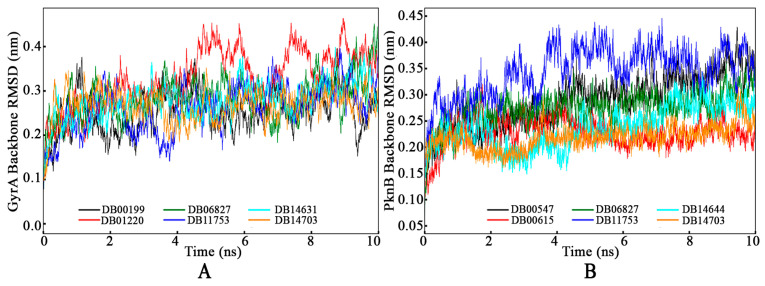
Protein backbone RMSD of (**A**) GyrA and (**B**) PknB bound with respective final drug molecules.

The backbone RMSD of GyrA and PknB bound with the respective proposed drug molecules was calculated throughout the MD simulation timeframe, and the results are presented in Figure 6. The GyrA backbone RMSD (Figure 6A) exhibited consistent behavior when bound with most of the proposed drug molecules, maintaining stability over the simulation period, except DB01220 and DB11753. Notably, the GyrA backbone bound with DB01220 demonstrated stability until approximately 4ns, following which a slight deviation from equilibrium was observed. Despite this deviation, no frames deviated beyond 0.465 nm, indicating a stable complex with a slightly higher RMSD. This observation suggests that the DB01220–GyrA complex exhibits overall stability with minor fluctuations, implying a relatively robust binding interaction that maintains structural coherence throughout the simulation duration. The GyrA backbone bound with DB11753 exhibited a slightly different deviation pattern than other complexes. However, it is noteworthy that this deviation followed a consistent trend, which could signify the system’s stability with potential conformational changes. The average GyrA backbone RMSD values were calculated to be 0.254 nm for DB00199, 0.328 nm for DB01220, 0.283 nm for DB06827, 0.262 nm for DB11753, 0.277 nm for DB14631, and 0.267 nm for DB14703. These low average backbone RMSD values across the different drug molecules indicate high consistency and stability within the systems. The minimal deviation observed in the GyrA backbone RMSD bound with drug molecule suggests that the complexes maintain a steady overall structure throughout the simulation, supporting the robustness and reliability of the drug–protein interactions.

The PknB backbone RMSD values bound with DB00547, DB00615, DB06827, DB11753, DB14644, and DB14703 were calculated from a 10 ns MD simulation period and depicted in Figure 6B. Across all complexes, the PknB backbone RMSD remained consistent and stable throughout the simulation. Specifically, the PknB backbone in the complex with DB11753 exhibited a slight initial deviation before reaching consistency at approximately 0.4 nm RMSD. By analyzing the difference between the maximum and minimum PknB backbone RMSD values, 0.429 nm for DB00547, 0.320 nm for DB00615, 0.364 nm for DB06827, 0.446 nm for DB11753, 0.342 nm for DB14644, and 0.316 nm for DB14703), insights into the extent of deviation during the simulation were obtained. Notably, no frames exceeded a deviation of 0.430 nm, indicating a high level of steadiness within all complexes. These observations clearly point towards achieving stability in all the PknB–drug complexes, emphasizing the reliability and consistency of the molecular interactions within these systems.

##### Ligand RMSD

**Figure 7:**
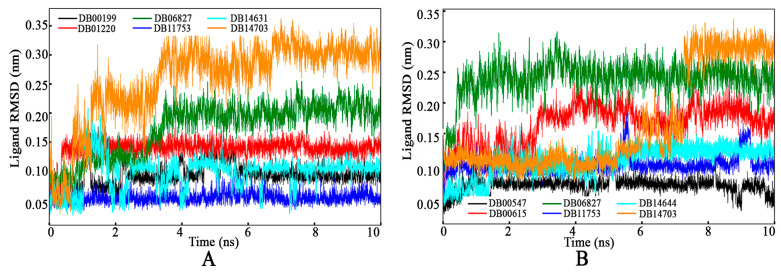
Ligand RMSD of proposed drug molecules bound with (**A**) Gyrase A and (**B**) PknB.

The dynamic behavior of each selected drug molecule within the active site cavity was investigated using MD simulation trajectories, and the ligand RMSD values were calculated and depicted in Figure 7. Throughout the simulations, the deviations of DB11753 and DB14703 remained consistent when bound to both GyrA and PknB. Specifically, DB14703 bound with PknB exhibited consistent behavior from the beginning to the end within the GyrA binding site. Interestingly, this molecule remained stable until approximately 6 ns, after which the RMSD increased to around 0.3 nm before stabilizing again. In contrast, the other molecules bound to either GyrA or PknB demonstrated stable conformational changes within their respective active sites throughout the simulation period. These findings indicate the structural dynamics and stability of the drug molecules within the binding sites of GyrA and PknB, highlighting their ability to maintain stable interactions and conformations conducive to effective binding and potential therapeutic efficacy.

#### 3.4.2. Root-Means-Square Fluctuation

**Figure 8:**
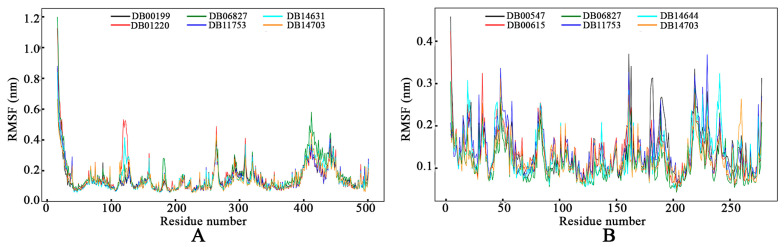
Root-mean-square fluctuation of (**A**) DNA GyrA and (**B**) PknB bound with respective proposed drug molecules.

In assessing the stability of the protein–ligand complexes during MD simulations, the dynamic behavior of individual amino acid residues was scrutinized through RMSF analyses, as presented in Figure 8. For GyrA and PknB, the RMSF values displayed systematic fluctuations without any irregular deviations, indicating a consistent and strong association between the proteins and ligands throughout the simulation period. The average RMSF values for GyrA were calculated to be 0.146 nm for DB00199, 0.163 nm for DB01220, 0.171 nm for DB06827, 0.146 nm for DB11753, 0.149 nm for DB14631, and 0.142 nm for DB14703. Similarly, the average RMSF values for PknB were observed to be 0.142 nm for DB00547, 0.134 nm for DB00615, 0.113 nm for DB06827, 0.142 nm for DB11753, 0.131 nm for DB14644, and 0.124 nm for DB14703. These consistent and relatively low RMSF values for both GyrA and PknB indicate stable fluctuations and well-maintained interactions between the amino acids and the respective ligands, reinforcing the stability and robust binding affinity of the protein–ligand complexes observed during the MD simulations.

#### 3.4.3. Radius of Gyration

**Figure 9:**
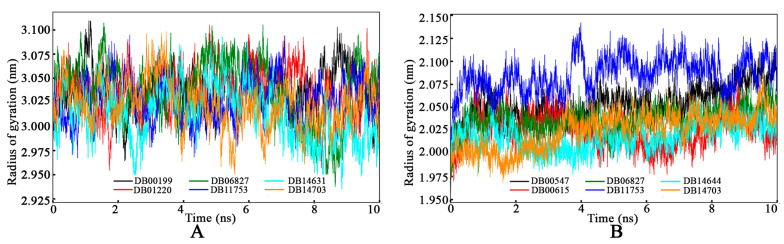
The radius of gyration of (**A**) GyrA and (**B**) PknB bound with proposed respective drug molecules.

The RoG values for GyrA and PknB bound with the final drug molecules were calculated and illustrated in Figure 9. The RoG values displayed consistent variations for GyrA when bound with DB00199, DB01220, DB06827, DB11753, DB14631, and DB14703, respectively. Conversely, the RoG of PknB exhibited relatively stable values around 2.025 nm, except for DB11753. Notably, around the 4 ns mark, PknB bound with DB11753 showcased a slightly higher deviation than the other complexes. Despite this variation, the RoG values for PknB bound with DB11753 did not exhibit extreme deviations, indicating overall stability. The consistent fluctuations in RoG for both GyrA and PknB suggest that all systems maintained their compactness and firmness within the dynamic molecular environment, confirming the robustness and structural reliability of the protein–ligand complexes throughout the simulation.

#### 3.4.4. Intermolecular Hydrogen Bond

**Figure 10:**
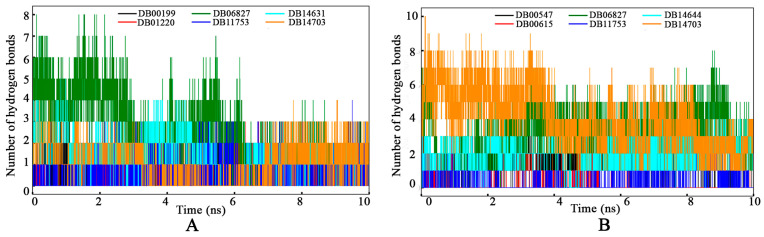
Intermolecular hydrogen bonds between (**A**) GyrA and (**B**) PknB with respective proposed drug molecules.

The hydrogen bonds formed between the proposed drugs and their respective protein receptors were calculated for each simulation frame and depicted in Figure 10. For the GyrA protein (Figure 10A), a high number of frames maintained their hydrogen bonding interactions with the drugs, signifying the robustness of these interactions. Notably, DB06827 was observed to form the highest number of hydrogen bonds (up to 8) with GyrA, indicating strong binding affinity and stability. Although a few frames did not demonstrate hydrogen bond formation, suggesting the potential involvement of non-hydrogen bond interactions in maintaining the complex stability. The MD simulation study observed a similar trend of hydrogen bonding interactions between the drug molecules and PknB. Specifically, DB14703 exhibited the maximum number of hydrogen bonds with PknB, highlighting its strong interaction with the receptor.

#### 3.4.5. Binding Free Energy Calculation Using MM-GBSA Approach

A total of 2000 frames were considered to calculate the free energy (ΔG_bind_) of the selected drug molecules towards GyrA and PknB. The results, including the calculated binding free energy values along with standard deviations, are presented in Table 4. A highly negative binding free energy signifies a strong affinity towards the receptor. Across the analyzed molecules, it is observed that all compounds demonstrated negative binding free energy ranging from −9 to −27 kcal/mol towards GyrA and −8 to −51 kcal/mol towards PknB. DB14703 exhibited the strongest binding affinity towards GyrA with a ΔG_bind_ of −27.750, while the highest affinity towards PknB was observed for DB14703 with a ΔG_bind_ of −51.510 kcal/mol. Conversely, the least binding affinity was observed for DB01220 and DB00615 towards GyrA and PknB, respectively, suggesting the potential need for optimization to enhance their binding affinities. These findings emphasize the importance of fine-tuning molecular structures to improve interactions and strengthen the binding affinity for effective GyrA and PknB therapeutic targeting.

#### 3.4.6. Free Energy Landscape

**Figure 11:**
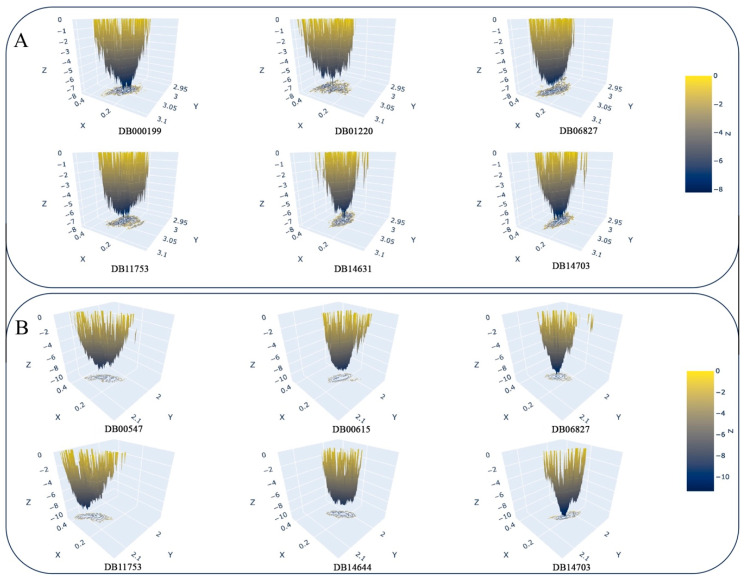
Free energy landscape of (**A**) GyrA and (**B**) PknB bound with final drug molecules.

Analyzing the FEL enables the identification of stable conformations, flexibility, transition pathways, and the impact of ligand binding, which is crucial for drug discovery efforts. In the FEL, low regions or valleys represent stable conformations, known as local minima. In contrast, high regions or peaks signify energy barriers and less stable conformations, referred to as transition states. The backbone RMSD and RoG values of GyrA and PknB bound with the final drug molecules were utilized to generate the FEL plots, depicted in Figure 11. The FEL plots illustrate that each plot displays stable conformations with multiple minima, indicating the presence of well-defined stable states. This suggests that the proposed drugs did not introduce instability in GyrA and PknB, underlining the maintenance of the constancy of the proteins in the presence of the drug molecules.

## 4. Conclusions

Drug repurposing is an excellent and powerful approach to finding the FDA drugs for any disease for which it was not invented. The current study was undertaken to screen the FDA-approved drug molecules against existing TB drugs through fingerprinting-based similarity search, multiple molecular docking studies, absolute binding affinity assessment, and MD simulation. Keeping in mind that the structurally similar structures can show the likely characteristics, highly structurally similar drugs to TB drugs were considered for further analysis with GyrA and PknB. Along with two different algorithms used in molecular docking, the ML-assisted absolute binding affinity was calculated. The aforementioned distinctive integrated drug-repurposing methodology effectively uncovers compounds exhibiting structural congruences with currently utilized tuberculosis pharmacotherapies, thereby facilitating novel pathways for repurposing initiatives. All the drug molecules retained after the similarity search were found to have at least some binding affinity towards GyrA and PknB. Six drug molecules for each of GyrA (DB00199, DB01220, DB06827, DB11753, DB14631, and DB14703) and PknB (DB00547, DB00615, DB06827, DB11753, DB14644, and DB14703) were found to be in the top 20 based on the binding energy from two docking approaches and absolute binding affinity. The binding interactions were also found to be comparable interactions between them. The MD simulation analyses strongly revealed no decline in the stability of any of the proteins due to the presence of the selected drug molecules. FEL was also suggested for stable conformation generation through several minima. A key finding of the study was the identification of three drug molecules, namely, DB06827, DB11753, and DB14703, which were common candidates for both GyrA and PknB. This shared selection suggests that these compounds possess characteristics that make them promising candidates for targeting both proteins simultaneously in the context of TB treatment. This dual-targeting strategy represents a significant innovation in TB treatment, potentially leading to more effective therapeutic outcomes. The consistent presence of these molecules across GyrA and PknB highlights their potential as versatile and effective therapeutic agents with dual targeting capabilities, offering a focused and efficient strategy for combating TB. Hence, it can be concluded that the proposed nine drug molecules can be re-used for treating and managing TB subjected to experimental validation.

## Figures and Tables

**Table 1:** The binding energy of selected drug molecules for GyrA and PknB from Autodock vina, PLANTS, and KDeep.

DrugBank ID	Binding Energy (kcal/mol)					
	Autodock Vina		PLANTS		K_Deep_	
	GyrA	PknB	GyrA	PknB	GyrA	PknB
DB00199	−8.000	−6.600	−81.060	−67.163	−9.650	−9.869
DB00547	−8.000	−8.000	−72.456	−78.860	−8.435	−9.961
DB00615	−8.400	−7.600	−69.230	−75.846	−11.475	−11.719
DB01220	−8.600	−7.200	−74.562	−70.409	−9.928	−10.194
DB06827	−8.400	−7.500	−85.054	−93.591	−11.251	−12.061
DB11753	−9.100	−8.500	−77.073	−79.248	−9.339	−9.995
DB14631	−8.300	−7.600	−77.792	−95.175	−9.003	−8.090
DB14644	−7.800	−8.100	−87.791	−96.450	−9.254	−10.889
DB14703	−9.100	−8.900	−81.491	−102.005	−10.626	−11.840

**Table 2:** Binding interacting amino acids between selected drugs and GyrA and PknB.

DrugBank IDs	Hydrogen Bonds	Hydrophobic Interactions	Salt Bridge
DNA GyrA			
DB00199	Asp94, Arg98	Ala40, Lys49, Val51, Ile92, Asp94, Ile181	Lys49, His52, Arg98
DB01220	Asp94, Arg98, Gln277, Asn279	Asp94, Arg98, Pro119	-
DB06827	Asp94, Arg98, Gln101, Trp103, Ser118, Pro119, Gly120, Pro124, Asn279	-	-
DB11753	Arg98, Gln277	Val97, Arg98, Pro124, Asn279	-
DB14631	Asp94, Ser95, Arg98, Arg98, Gln101, Gln101, Gln277, Val278	Gln277	-
DB14703	Asn115, Gly117, Ser307, Asp308, Gly311	Trp103, Pro119, Asp308, Leu312	-
Serine/threonine-protein kinase PknB			
DB00547	Val95, Ala142	Leu17, Val25, Thr99	
DB00615	Gly21, Asp138, Lys140, Asn143, Asp156, Ala180	Phe19	-
DB06827	Phe19, Gly21, Ser23, Glu59, Arg101, Asp102, Asp156	Met155	Asp102
DB11753	Leu17, Phe19, Arg101	Val25, Ala38, Val95, Val98, Asp156	-
DB14644	Ser23, Val95, Thr99, Ala142	Leu17, Thr99	Lys40
DB14703	Glu59, Asp138, Ala142, Gly158, Ile159	Leu17, Val25, Thr99	Lys140

**Table 3:** Statistical data from MD simulation of FDA-approved drugs bound with GyrA and PknB.

		DNA GyrA						Serine/Threonine-Protein Kinase PknB					
		G1	G2	G3	G4	G5	G6	S1	S2	G3	G4	S3	G6
Backbone RMSD (nm)	Max.	0.396	0.464	0.451	0.396	0.398	0.352	0.429	0.320	0.364	0.446	0.342	0.316
	Min.	0.000	0.001	0.000	0.000	0.001	0.000	0.000	0.000	0.000	0.000	0.000	0.000
	Avg.	0.254	0.328	0.283	0.262	0.277	0.267	0.289	0.228	0.275	0.343	0.238	0.218
Ligand RMSD (nm)	Max.	0.172	0.178	0.256	0.113	0.208	0.363	0.110	0.245	0.317	0.184	0.162	0.337
	Min.	0.001	0.000	0.001	0.000	0.000	0.000	0.001	0.000	0.001	0.000	0.001	0.000
	Avg.	0.093	0.139	0.173	0.054	0.097	0.257	0.064	0.164	0.239	0.098	0.104	0.163
RMSF (nm)	Max.	0.957	1.198	1.352	0.987	0.880	0.790	0.695	0.495	0.373	0.368	0.324	0.313
	Min.	0.063	0.067	0.060	0.061	0.059	0.057	0.050	0.051	0.043	0.067	0.047	0.047
	Avg.	0.146	0.163	0.171	0.146	0.149	0.142	0.142	0.134	0.113	0.142	0.131	0.124
RoG (nm)	Max.	3.110	3.105	3.107	3.095	3.093	3.098	2.115	2.082	2.078	2.142	2.064	2.084
	Min.	2.964	2.954	2.937	2.967	2.930	2.949	1.938	1.936	1.937	1.938	1.937	1.937
	Avg.	3.038	3.038	3.038	3.027	3.013	3.022	2.054	2.025	2.037	2.085	2.018	2.025

G1: DB00199; G2: DB01220; G3: DB06827; G4: DB11753; G5: DB14631; G6: DB14703; S1: DB00547; S2: DB00615; S3: DB14644; Min.: minimum; Max.: maximum; Avg.: average.

**Table 4:** The binding free energy of the selected drug molecules towards GyrA and PknB.

	Molecule	ΔG_bind_ (kcal/mol)	Standard Deviation
GyrA	DB00199	−21.450	±4.050
	DB01220	−9.650	±4.630
	DB06827	−20.130	±5.050
	DB11753	−19.540	±3.870
	DB14631	−20.070	±4.800
	DB14703	−27.750	±4.760
PknB	DB00547	−25.720	±3.980
	DB00615	−8.580	±3.770
	DB06827	−19.570	±5.880
	DB11753	−24.750	±3.850
	DB14644	−36.460	±6.270
	DB14703	−51.510	±14.150

## Data Availability

No new data were created or analyzed in this study. Data sharing is not applicable to this article.

## Supplementary Materials

The following supporting information can be downloaded at: https://www.mdpi.com/article/10.3390/tropicalmed9120288/s1, Table S1: SMILES representation and binding energy from Autodock vina, PLANTS and KDeep of TB drug molecules; Table S2: SMILES representation and binding energy from Autodock vina, PLANTS and KDeep of drug molecules retained after similarity search.
