# Supplementary material for: Identification of Anti-Tuberculosis Drugs Targeting DNA Gyrase A and Serine/Threonine Protein Kinase PknB: A Machine Learning-Assisted Drug-Repurposing Approach
Source: Trop Med Infect Dis. 2024 Nov 25;9(12):288. doi: 10.3390/tropicalmed9120288 (PMC11679129; doi:10.3390/tropicalmed9120288)
Supplement: Supplementary file 1 [file tropicalmed-09-00288-s001.zip › tropicalmed-3182242-supplementary.pdf]

Tropical Medicine and Infectious Disease

MDPI

Supplementary Materials

Table S1. SMILES representation and binding energy from Autodock vina, PLANTS and K_{Deep} of TB drug molecules.

ID	SMILES	Binding energy (kcal/mol)					
		Autodock vina		PLANTS		K_{Deep}	
		GyrA	PknB	GyrA	PknB	GyrA	PknB
**DB00218**	[H][C@]12CN(C[C@@]1([H])NCCC2)C1=C(F)C=C2C(=O)C(=CN(C3CC3)C2=C1OC)C(O)=O	-8.300	-8.800	-62.405	-76.153	-8.939	-8.908
DB00233	NC1=CC(O)=C(C=C1)C(O)=O	-5.400	-5.200	-62.698	-60.551	-4.276	-5.109
DB00260	N[C@@H]1CONC1=O	-4.400	-4.300	-46.397	-52.430	-4.182	-3.452
DB00314	[H][C@@]1(CCN=C(N)N1)[C@]1([H])NC(=O)\C(NC(=O)[C@H](CNC(=O)C[C@@H](N)CCCN)NC(=O)[C@H](C)NC(=O)[C@@H](N)CNC1=O)=C/NC(N)=O.[H][C@@]1(CCN=C(N)N1)[C@]1([H])NC(=O)\C(NC(=O)[C@H](CNC(=O)C[C@@H](N)CCCN)NC(=O)[C@H](CO)NC(=O)[C@@H](N)CNC1=O)=C/NC(N)=O	-7.900	-8.600	-94.843	-88.794	-9.993	-11.613
DB00330	CC[C@@H](CO)NCCN[C@@H](CC)CO	-4.500	-4.500	-69.648	-72.622	-3.516	-6.082
DB00339	NC(=O)C1=NC=CN=C1	-5.000	-4.700	-53.815	-53.882	-5.015	-4.844
DB00479	NCC[C@H](O)C(=O)N[C@@H]1C[C@H](N)[C@@H](O[C@H]2O[C@H](CN)[C@@H](O)[C@H](O)[C@H]2O)[C@H](O)[C@H]1O[C@H]1O[C@H](CO)[C@@H](O)[C@H](N)[C@H]1O	-7.000	-7.000	-90.865	-92.938	-7.436	-11.303
DB00537	OC(=O)C1=CN(C2CC2)C2=CC(N3CCNCC3)=C(F)C=C2C1=O	-7.300	-8.200	-65.115	-81.651	-6.995	-8.765
DB00601	CC(=O)NC[C@H]1CN(C(=O)O1)C1=CC(F)=C(C=C1)N1CCOCC1	-7.100	-7.700	-65.659	-82.384	-5.506	-8.076
DB00609	CCC1=NC=CC(=C1)C(N)=S	-4.800	-4.900	-64.515	-63.238	-5.150	-7.227
DB00620	[H][C@@]12C[C@@H](O)[C@](O)(C(=O)CO)[C@@]1(C)C[C@H](O)[C@@]1(F)[C@@]2([H])CCC2=CC(=O)C=C[C@]12C	-7.700	-7.900	-70.180	-74.703	-7.584	-8.704
DB00860	[H][C@@]12CC[C@](O)(C(=O)CO)[C@@]1(C)C[C@H](O)[C@@]1([H])[C@@]2([H])CCC2=CC(=O)C=C[C@]12C	-8.200	-7.300	-75.036	-79.396	-8.132	-7.399
DB00951	NNC(=O)C1=CC=NC=C1	-5.700	-4.900	-57.208	-55.876	-5.664	-5.810
DB00959	[H][C@@]12CC[C@](O)(C(=O)CO)[C@@]1(C)C[C@H](O)[C@@]1([H])[C@@]2([H])C[C@H](C)C2=CC(=O)C=C[C@]12C	-7.500	-7.600	-76.234	-86.320	-8.703	-8.504
DB01041	O=C1N(C2CCC(=O)NC2=O)C(=O)C2=CC=CC=C12	-7.000	-7.700	-66.875	-66.307	-4.483	-6.155

DB01044	COC1=C2N(C=C(C(O)=O)C(=O)C2=CC(F)=C1N1CCNC(C)C1)C1CC1	-7.400	-8.200	-60.494	-72.812	-7.717	-8.629
DB01045	CO[C@H]1\C=C\O[C@@]2(C)OC3=C(C2=O)C2=C(O)C(\C=N\N4CCN(C)CC4)=C(NC(=O)\C(C)=C/C=C/[C@H](C)[C@H](O)[C@@H](C)[C@@H](O)[C@@H](C)[C@H](OC(C)=O)[C@@H]1C)C(O)=C2C(O)=C3C	-7.600	-7.600	-25.791	-22.657	-10.647	-10.657
DB01060	[H][C@]12SC(C)(C)[C@@H](N1C(=O)[C@H]2NC(=O)[C@H](N)C1=CC=C(O)C=C1)C(O)=O	-7.200	-7.400	-78.906	-79.996	-6.015	-7.741
DB01082	CN[C@H]1[C@H](O)[C@@H](O)[C@H](CO)O[C@H]1O[C@H]1[C@H](O[C@H]2[C@H](O)[C@@H](O)[C@H](NC(N)=N)[C@@H](O)[C@@H]2NC(N)=N)O[C@@H](C)[C@]1(O)C=O	-7.500	-7.600	-82.734	-88.497	-7.345	-8.039
DB01137	C[C@H]1COC2=C3N1C=C(C(O)=O)C(=O)C3=CC(F)=C2N1CCN(C)CC1	-7.700	-8.400	-68.585	-80.703	-6.540	-8.134
DB01165	CC1COC2=C3N1C=C(C(O)=O)C(=O)C3=CC(F)=C2N1CCN(C)CC1	-7.500	-7.600	-67.806	-79.885	-6.527	-7.513
DB01172	NC[C@H]1O[C@H](O[C@@H]2[C@@H](N)C[C@@H](N)[C@H](O[C@H]3O[C@H](CO)[C@@H](O)[C@H](N)[C@H]3O)[C@H]2O)[C@H](O)[C@@H](O)[C@@H]1O	-7.300	-7.100	-81.719	-81.336	-6.652	-10.441
DB01201	CO[C@H]1\C=C\O[C@@]2(C)OC3=C(C2=O)C2=C(C(O)=C3C)C(O)=C(NC(=O)\C(C)=C/C=C/[C@H](C)[C@H](O)[C@@H](C)[C@@H](O)[C@@H](C)[C@H](OC(C)=O)[C@@H]1C)C(\C=N\N1CCN(CC1)C1CCCC1)=C2O	-8.000	-7.600	-47.035	-43.649	-11.759	-11.949
DB01211	[H][C@@]1(C[C@@](C)(OC)[C@@H](O)[C@H](C)O1)O[C@H]1[C@H](C)[C@@H](O[C@]2([H])O[C@H](C)C[C@@H]([C@H]2O)N(C)C)[C@@](C)(C[C@@H](C)C(=O)[C@H](C)[C@@H](O)[C@](C)(O)[C@@H](CC)OC(=O)[C@@H]1C)OC	-7.000	-6.500	-57.555	-51.920	-9.509	-10.044
DB01234	[H][C@@]12C[C@@H](C)[C@](O)(C(=O)CO)[C@@]1(C)C[C@H](O)[C@@]1(F)[C@@]2([H])CCC2=CC(=O)C=C[C@]12C	-8.100	-7.600	-73.186	-77.178	-7.463	-8.754
DB01380	[H][C@@]12CC[C@](O)(C(=O)COC(C)=O)[C@@]1(C)CC(=O)[C@@]1([H])[C@@]2([H])CCC2=CC(=O)CC[C@]12C	-7.600	-7.900	-75.326	-83.390	-8.501	-8.596
DB05154	[O-][N+](=O)C1=CN2C[C@@H](COC2=N1)OCC1=CC=C(OC(F)(F)F)C=C1	-7.200	-7.900	-75.325	-82.749	-6.869	-7.677

DB08903	COC1=NC2=C(C=C(Br)C=C2)C=C1[C@@H](C1=CC=CC=C1)[C@@](O)(CCN(C)C)C1=CC=CC2=C1C=CC=C2	-7.300	-7.400	-64.474	-65.515	-8.606	-8.916
DB11637	C[C@]1(COC2=CC=C(C=C2)N2CCC(CC2)OC2=CC=C(OC(F)(F)F)C=C2)CN2C=C(N=C2O1)[N+]([O-])=O	-9.100	-7.900	-79.529	-84.174	-9.183	-9.113
DB12667	CCCC1=CC(=CC=N1)C(N)=S	-4.900	-5.100	-66.550	-68.734	-4.839	-7.381

Table S2. SMILES representation and binding energy from Autodock vina, PLANTS and K_{Deep} of drug molecules retained after similarity search.

ID	SMILES	Binding energy (kcal/mol)					
		Autodock vina		PLANTS		K_{Deep}	
		GyrA	PknB	GyrA	PknB	GyrA	PknB
DB00199	CC[C@H]1OC(=O)[C@H](C)[C@@H](O[C@H]2C[C@@](C)(OC)[C@@H](O)[C@H](C)O2)[C@H](C)[C@@H](O[C@@H]2O[C@H](C)C[C@@H]([C@H]2O)N(C)C)[C@](C)(O)C[C@@H](C)C(=O)[C@H](C)[C@@H](O)[C@]1(C)O	-8.000	-6.600	-81.060	-67.163	-9.650	-9.869
DB00207	CC[C@H]1OC(=O)[C@H](C)[C@@H](O[C@H]2C[C@@](C)(OC)[C@@H](O)[C@H](C)O2)[C@H](C)[C@@H](O[C@@H]2O[C@H](C)C[C@@H]([C@H]2O)N(C)C)[C@](C)(O)C[C@@H](C)CN(C)[C@H](C)[C@@H](O)[C@]1(C)O	-6.700	-5.900	-57.677	-51.037	-9.375	-9.407
DB00223	[H][C@@]12C[C@H](C)[C@](O)(C(=O)CO)[C@@]1(C)C[C@H](O)[C@@]1(F)[C@@]2([H])C[C@H](F)C2=CC(=O)C=C[C@]12C	-7.800	-7.300	-68.190	-67.381	-7.304	-8.974
DB00244	NC1=CC(C(O)=O)=C(O)C=C1	-5.700	-5.200	-66.766	-63.211	-4.883	-5.253
DB00365	CC1CN(CCN1)C1=C(F)C(C)=C2C(=O)C(=CN(C3CC3)C2=C1)C(O)=O	-7.400	-8.500	-65.156	-83.893	-6.514	-8.715

DB00415	[H][C@]12SC(C)(C)[C@@H](N1C(=O)[C@H]2NC(=O)[C@H](N)C1=CC=CC=C1)C(O)=O	-6.800	-7.000	-76.270	-79.090	-5.460	-7.340
DB00452	NC[C@@H]1O[C@H](O[C@@H]2[C@@H](CO)O[C@@H](O[C@@H]3[C@@H](O)[C@H](N)C[C@H](N)[C@H]3O[C@H]3O[C@H](CN)[C@@H](O)[C@H](O)[C@H]3N)[C@@H]2O)[C@H](N)[C@@H](O)[C@@H]1O	-7.200	-6.700	-91.452	-89.701	-9.013	-9.112
DB00547	[H][C@@]12C[C@@H](C)[C@H](C(=O)CO)[C@@]1(C)C[C@H](O)[C@@]1(F)[C@@]2([H])CCC2=CC(=O)C=C[C@]12C	-8.000	-8.000	-72.456	-78.860	-8.435	-9.961
DB00596	[H][C@@]12C[C@H](C)[C@](O)(C(=O)CCl)[C@@]1(C)C[C@H](O)[C@@]1(F)[C@@]2([H])C[C@H](F)C2=CC(=O)C=C[C@]12C	-8.200	-7.600	-62.235	-65.030	-8.304	-7.614
DB00615	CO[C@H]1\C=C\O[C@@]2(C)OC3=C(C2=O)C2=C(C(O)=C3C)C(=O)C(NC(=O)\C(C)=C/C=C/[C@H](C)[C@H](O)[C@@H](C)[C@@H](O)[C@@H](C)[C@H](OC(C)=O)[C@@H]1C)=C1NC3(CCN(CC3)CC(C)C)N=C21	-8.400	-7.600	-69.230	-75.846	-11.475	-11.719
DB00635	[H][C@@]12CC[C@](O)(C(=O)CO)[C@@]1(C)CC(=O)[C@@]1([H])[C@@]2([H])CCC2=CC(=O)C=C[C@]12C	-7.900	-7.700	-72.388	-86.097	-7.371	-7.168
DB00663	[H][C@@]12C[C@@H](C)[C@](O)(C(=O)CO)[C@@]1(C)C[C@H](O)[C@@]1(F)[C@@]2([H])C[C@H](F)C2=CC(=O)C=C[C@]12C	-8.000	-7.300	-72.039	-78.742	-7.479	-8.265

DB00684	NC[C@H]1O[C@H](O[C@@H]2[C@@H](N)C[C@@H](N)[C@H](O[C@H]3O[C@H](CO)[C@@H](O)[C@H](N)[C@H]3O)[C@H]2O)[C@H](N)C[C@@H]1O	-7.700	-6.900	-83.117	-92.343	-7.548	-7.702
DB00741	[H][C@@]12CC[C@](O)(C(=O)CO)[C@@]1(C)C[C@H](O)[C@@]1([H])[C@@]2([H])CCC2=CC(=O)CC[C@]12C	-7.600	-7.200	-70.017	-77.280	-7.799	-7.770
DB00778	CC[C@H]1OC(=O)[C@H](C)[C@@H](O[C@H]2C[C@@](C)(OC)[C@@H](O)[C@H](C)O2)[C@H](C)[C@@H](O[C@@H]2O[C@H](C)C[C@@H]([C@H]2O)N(C)C)[C@](C)(O)C[C@@H](C)C(=NOCOCCOC)[C@H](C)[C@@H](O)[C@]1(C)O	-7.000	-6.500	-93.681	-82.140	-11.576	-11.486
DB00873	[H][C@@]12CC[C@](O)(C(=O)OCCl)[C@@]1(C)C[C@H](O)[C@@]1([H])[C@@]2([H])CCC2=CC(=O)C=C[C@]12C	-8.200	-7.200	-75.500	-80.905	-7.596	-7.305
DB00896	[H][C@@]12C[C@@H](C)[C@](C)(C(=O)CC)[C@@]1(C)C[C@H](O)[C@@]1([H])[C@@]2([H])CCC2=CC(=O)C=C[C@]12C	-8.300	-7.200	-67.901	-65.568	-8.614	-9.116
DB00978	CCN1C=C(C(O)=O)C(=O)C2=CC(F)=C(N3CCNC(C)C3)C(F)=C12	-7.800	-8.200	-65.252	-80.529	-7.693	-8.572
DB01013	[H][C@@]12C[C@H](C)[C@](OC(=O)CC)(C(=O)CCl)[C@@]1(C)C[C@H](O)[C@@]1(F)[C@@]2([H])CCC2=CC(=O)C=C[C@]12C	-7.300	-6.800	-63.858	-52.694	-8.437	-9.007
DB01059	CCN1C=C(C(O)=O)C(=O)C2=CC(F)=C(C=C12)N1CCNCC1	-6.900	-7.900	-62.747	-80.945	-6.563	-8.383

DB01220	CO[C@H]1\C=C\O[C@@]2(C)OC3=C(C)C(O)=C4C(O)=C(NC(=O)\C(C)=C/C=C/[C@H](C)[C@H](O)[C@@H](C)[C@@H](O)[C@@H](C)[C@H](OC(C)=O)[C@@H]1C)C1=C(N=C5C=C(C)C=CN15)C4=C3C2=O	-8.600	-7.200	-74.562	-70.409	-9.928	-10.194
DB01222	[H][C@@]12C[C@H]3OC(CCC)O[C@@]3(C(=O)CO)[C@@]1(C)C[C@H](O)[C@@]1([H])[C@@]2([H])CCC2=CC(=O)C=C[C@]12C	-8.400	-7.100	-77.144	-76.961	-7.791	-8.224
DB01260	[H][C@@]12C[C@H]3OC(C)(C)O[C@@]3(C(=O)CO)[C@@]1(C)C[C@H](O)[C@@]1([H])[C@@]2([H])CCC2=CC(=O)C=C[C@]12C	-8.300	-6.900	-70.242	-75.453	-7.256	-7.122
DB01421	NC[C@@H]1O[C@H](O[C@@H]2[C@@H](CO)O[C@@H](O[C@@H]3[C@@H](O)[C@H](N)C[C@H](N)[C@H]3O[C@H]3O[C@H](CO)[C@@H](O)[C@H](O)[C@H]3N)[C@@H]2O)[C@H](N)[C@@H](O)[C@@H]1O	-6.700	-6.700	-89.077	-90.275	-7.586	-9.108
DB03615	[H][C@@]1(O[C@@H]2[C@@H](O)[C@H](N)C[C@H](N)[C@H]2O[C@H]2O[C@H](CN)[C@@H](O)[C@H](O)[C@H]2N)O[C@H](CO)[C@@H](O)[C@H]1O	-6.700	-6.900	-79.932	-87.888	-6.967	-9.054
DB06827	[H][C@@]1(C[C@H](O)NC(=N)N1)[C@]1([H])NC(=O)\C(NC(=O)[C@H](CO)NC(=O)[C@H](CO)NC(=O)[C@H](CNC1=O)NC(=O)C[C@@H](N)CCCN)=C\NC(N)=O	-8.400	-7.500	-85.054	-93.591	-11.251	-12.061
DB08910	NC1=C2C(=O)N(C3CCC(=O)NC3=O)C(=O)C2=CC=C1	-7.400	-7.400	-65.938	-73.151	-4.979	-6.357

DB09047	[H][C@]12CN(C[C@]1([H])OCCN2)C1=C(F)C=C2C(=O)C(=CN(C3CC3)C2=C1C#N)C(O)=O	-7.200	-8.700	-65.853	-77.694	-7.575	-9.465
DB09378	[H][C@@]12CC[C@](O)(C(=O)CO)[C@@]1(C)C[C@H](O)[C@@]1([H])[C@@]2([H])C[C@H](F)C2=CC(=O)C=C[C@]12C	-7.900	-7.700	-76.795	-84.216	-7.648	-7.556
DB11512	CN[C@H]1[C@H](O)[C@@H](O)[C@H](CO)O[C@H]1O[C@H]1[C@H](O[C@H]2[C@H](O)[C@@H](O)[C@H](NC(N)=N)[C@@H](O)[C@@H]2NC(N)=N)O[C@@H](C)[C@]1(O)CO	-7.200	-6.500	-70.167	-73.382	-6.971	-8.138
DB11750	[H][C@@]12C[C@H](C)[C@](O)(C(=O)CCl)[C@@]1(C)C[C@H](O)[C@@]1(F)[C@@]2([H])CCC2=CC(=O)C=C[C@]12C	-7.500	-7.300	-66.217	-70.246	-8.306	-8.832
DB11753	CO[C@H]1\C=C\O[C@@]2(C)OC3=C(C)C(O)=C4C(O)=C(NC(=O)\C(C)=C/C=C/[C@H](C)[C@H](O)[C@@H](C)[C@@H](O)[C@@H](C)[C@H](OC(C)=O)[C@@H]1C)C=C(O)C4=C3C2=O	-9.100	-8.500	-77.073	-79.248	-9.339	-9.995
DB12952	[H][C@@]12CC[C@](O)(C(=O)CO)[C@@]1(C)CC(=O)[C@@]1([H])[C@@]2([H])C[C@H](C)C2=CC(=O)C=C[C@]12C	-7.600	-7.700	-73.626	-79.813	-7.422	-8.459
DB13158	[H][C@]1(C)C[C@@]2([H])[C@]3([H])CCC4=CC(=O)C=C[C@]4(C)[C@@]3(F)C(=O)C[C@]2(C)[C@@]1(O)C(=O)CCl	-8.400	-6.900	-67.015	-68.502	-7.973	-6.625
DB13270	NC[C@@H]1CC[C@@H](N)[C@@H](O[C@@H]2[C@@H](N)C[C@@H](N)[C@H](O[C@H]3O[C@H](CO)[C@@H	-7.400	-6.600	-79.404	-76.677	-7.164	-8.479

	](O)[C@H](N)[C@H]3O)[C@H]2O)O1						
DB14539	[H][C@@]12CC[C@](O)(C(=O)COC(C)=O)[C@@]1(C)C[C@H](O)[C@@]1([H])[C@@]2([H])CCC2=CC(=O)CC[C@]12C	-7.500	-7.900	-72.887	-81.906	-8.180	-8.693
DB14631	[H][C@@]12CC[C@](O)(C(=O)COP(O)(O)=O)[C@@]1(C)C[C@H](O)[C@@]1([H])[C@@]2([H])CCC2=CC(=O)C=C[C@]12C	-8.300	-7.600	-77.792	-95.175	-9.003	-8.090
DB14632	[H][C@@]12CC[C@](O)(C(=O)COC(=O)CC(C)(C)C)[C@@]1(C)C[C@H](O)[C@@]1([H])[C@@]2([H])CCC2=CC(=O)C=C[C@]12C	-7.700	-7.600	-82.890	-78.102	-7.890	-9.579
DB14643	[H][C@@]12CC[C@](OC(=O)CC)(C(=O)COC(C)=O)[C@@]1(C)C[C@H](O)[C@@]1([H])[C@@]2([H])C[C@H](C)C2=CC(=O)C=C[C@]12C	-7.800	-6.800	-68.293	-69.797	-9.075	-9.229
DB14644	[H][C@@]12CC[C@](O)(C(=O)COC(=O)CCC(O)=O)[C@@]1(C)C[C@H](O)[C@@]1([H])[C@@]2([H])C[C@H](C)C2=CC(=O)C=C[C@]12C	-7.800	-8.100	-87.791	-96.450	-9.254	-10.889
DB14646	[H][C@@]12CC[C@](O)(C(=O)COC(C)=O)[C@@]1(C)CC(=O)[C@@]1([H])[C@@]2([H])CCC2=CC(=O)C=C[C@]12C	-8.200	-7.900	-70.615	-83.635	-8.369	-8.308
DB14649	[H][C@@]12C[C@@H](C)[C@](O)(C(=O)COC(C)=O)[C@@]1(C)C[C@H](O)[C@@]1(F)[C@@]2([H])CCC2=CC(=O)C=C[C@]12C	-8.800	-7.600	-70.574	-82.420	-8.584	-8.443
DB14669	[H][C@@]12C[C@H](C)[C@](O)(C(=O)COP(O)(O)=O)[C@@]1(C)C[C@H](O)[C@@]	-7.600	-7.400	-71.227	-78.793	-8.480	-9.536

	1(F)[C@@]2([H])CCC2=CC(=O)C=C[C@]12C						
DB14703	[H][C@@]12C[C@@H](C)[C@](O)(C(=O)COC(=O)C3=CC=CC(=C3)S(O)(=O)=O)[C@@]1(C)C[C@H](O)[C@@]1(F)[C@@]2([H])CCC2=CC(=O)C=C[C@]12C	-9.100	-8.900	-81.491	-102.005	-10.626	-11.840
DB15566	[H][C@@]12CC[C@](O)(C(=O)COC(C)=O)[C@@]1(C)C[C@]([H])(O)[C@@]1([H])[C@@]2([H])CCC2=CC(=O)C=C[C@]12C	-7.700	-7.400	-73.601	-76.397	-7.396	-8.225
